# Improved lipids, diastolic pressure and kidney function are potential contributors to familial longevity: a study on 60 Chinese centenarian families

**DOI:** 10.1038/srep21962

**Published:** 2016-02-25

**Authors:** Yong-Han He, Shao-Yan Pu, Fu-Hui Xiao, Xiao-Qiong Chen, Dong-Jing Yan, Yao-Wen Liu, Rong Lin, Xiao-Ping Liao, Qin Yu, Li-Qin Yang, Xing-Li Yang, Ming-Xia Ge, Ying Li, Jian-Jun Jiang, Wang-Wei Cai, Qing-Peng Kong

**Affiliations:** 1 State Key Laboratory of Genetic Resources and Evolution, Kunming Institute of Zoology, the Chinese Academy of Sciences, Kunming 650223, China; 2 Department of Biochemistry and Molecular Biology, Hainan Medical College, Haikou 571199, China; 3 KIZ/CUHK Joint Laboratory of Bioresources and Molecular Research in Common Diseases, Kunming 650223, China; 4 Department of Biology, Hainan Medical College, Haikou 571199, China; 5 Department of Neurology, the Affiliated Hospital of Hainan Medical College, Haikou 571199, China; 6 Kunming College of Life Science, University of Chinese Academy of Sciences, Beijing 100049, China; 7 Farm Animal Genetic Resources Exploration and Innovation Key Laboratory of Sichuan Province, Sichuan Agricultural University, Chengdu 611130, China

## Abstract

Centenarians are a good healthy aging model. Interestingly, centenarians’ offspring are prone to achieve longevity. Here we recruited 60 longevity families and investigated the blood biochemical indexes of family members to seek candidate factors associated with familial longevity. First, associations of blood indexes with age were tested. Second, associations of blood parameters in centenarians (CEN) with their first generation of offspring (F1) and F1 spouses (F1SP) were analyzed. Third, genes involved in regulating target factors were investigated. We found that total cholesterol (TC) and triglyceride (TG) increased with age (20–80 years), but decreased in CEN. Similarly, blood urea nitrogen (BUN) and blood creatinine (BCr) increased with age (20–80 years), but were maintained on a plateau in CEN. Importantly, we first revealed dual changes in blood pressure, i.e., decreased diastolic blood pressure but increased systolic blood pressure in CEN, which associated with altered *CST3* expression. Genetic analysis revealed a significant association of blood uric acid (BUA) and BCr in CEN with F1 but not with F1SP, suggesting they may be heritable traits. Taken together, our results suggest serum lipids, kidney function and especially diastolic pressure rather than systolic pressure were improved in CEN or their offspring, suggesting these factors may play an important role in familial longevity.

Centenarians live to or beyond the age of 100 years, surpassing the current human life expectancy with about 20–25 years. Several cross-sectional and follow-up studies investigated the centenarians and revealed that they usually manage to escape or delay major age-related diseases, especially those with high morbidity and mortality, such as cardiovascular disease, neurodegeneration and cancers^1^,^2^,^3^,^4^. Therefore, centenarians are considered as a good model for healthy aging study. Interestingly, the offspring of longevity subjects can also usually obtain similar survival advantage of postponing age-related diseases^3^,^5^,^6^,^7^,^8^,^9^, suggesting the involvement of heritable factors in the familial longevity. Indeed, multiple determinants on human lifespan including various environmental and genetic factors have been identified via longevity population studies^10^,^11^. It has been shown that age at death in adulthood has a heritability of approximately 25%^12^. However, the longevity heritability increases with greater age with the estimated heritability of living to 100 was 33% in women and 48% in men^13^, therefore the genetic mechanisms of longevity seem to be age-dependent and remain to be further explored. Moreover, it is feasible to find beneficial factors in longevity subject so as to help the general elderly to improve their healthy status.

We previously investigated 535 centenarians and identified some protective or detrimental risk variants^14^,^15^. Nevertheless, most of the longevity studies including ours were case-control designs comprised of centenarians and younger controls, by which we can just explore factors for longevity of centenarians, but it is hard to identify those with familial longevity. Instead, longevity families comprised of centenarians, centenarians’ offspring, sons- or daughters-in-law, constitute a good possibility for identifying factors for familial longevity. However, familial longevity studies are quite limited, which may result from the difficulties in sample recruiting due to wide geographic distribution and very low frequency for centenarian families.

Last year, we managed to recruit 60 longevity families from Hainan province, a well-known longevity region in China, and performed a complete physical examination on all subjects. Based on this population, we found that the thyroid function was associated with longevity and could be heritable^16^. In this study we expanded the study by investigating associations of the rest blood parameters with age, and associations between generations, aiming to seek candidate factors associated with familial longevity. Finally, we found that serum lipids and renal function were improved in centenarians and suggested the renal function to be a heritable factor. Of notice is that we revealed for the first time that diastolic pressure rather than systolic pressure were improved in centenarians, being an important and favorable contributor to longevity.

## Methods

### Subjects

60 longevity families (a total of 206 subjects) consisting of 61 centenarians, 63 first generation of offspring (F1), 47 spouses of F1 (F1SP), 25 second generation of offspring (F2) and 10 spouses of F2 (F2SP) were recruited from Hainan province of China in July, 2014. The average ages are 102.70, 62.23, 59.90, 31.87, and 31.11 years, respectively. All subjects are the Han nationality. Of them, 40 families were available to test the genetic correlations of blood biochemical indexes.

### Blood analysis

All subjects were invited to participate in a physical examination. Clinical biochemical parameters including total cholesterol (TC), high density lipoprotein-cholesterol (HDL-C), low density lipoprotein-cholesterol (LDL-C), triglyceride (TG), blood glucose (BG), thyroid-stimulating hormone (TSH), triiodothyronine (T3), thyroxine (T4), free T3, free T4, alanine aminotransferase (ALT), aspartate aminotransferase (AST), total protein (TP), albumin, globulin, total bilirubin, direct bilirubin, indirect bilirubin, blood urea nitrogen (BUN), blood uric acid (BUA) and blood creatinine (BCr) were determined following standard laboratory procedures. Blood pressure was measured using a standard mercury sphygmomanometer on the right arm after at least 10 min of rest. History of severe diseases in cardiovascular, pulmonary, digestive, urinary, and nervous systems was recorded according to centenarian self- or their family-reporting data. The study protocol was approved by the Ethics Committee at Kunming Institute of Zoology, Chinese Academy of Sciences. Written informed consent was obtained from each of the participants. All methods were carried out in “accordance” with the approved guidelines.

### RNA extraction and RNA sequencing analysis

63 samples consisting of 27 centenarians, 18 F1 and 18 F1SP were selected to perform RNA-sequencing (RNA-seq). Total RNA was isolated from 3 mL of fresh blood using the Trizol reagent (Invitrogen). The RNA-seq was performed at the Beijing Genomics Institute at Shenzhen according to the manufacturer’s protocol. To estimate the level of gene expression, the reads count was transformed into fragments per kilo bases per million mapped reads (FPKM)^17^ which reflects the abundance of the gene expression. Of the 63 samples, 13 longevity families were available for analyzing the association of gene expression between the CEN and F1 as well as F1SP (supplemental table 1).

### Data extraction of genes from the RNA-seq dataset

For those candidate blood indexes, we systematically searched the National Center for Biotechnology Information (NCBI, http://www.ncbi.nlm.nih.gov/), Genecards (http://www.genecards.org/) and Genome-Wide Association Studies (GWAS) Catalog (http://www.genome.gov/gwastudies/) databases and collected related genes. Genes involved in lipid metabolism were retrieved from the Transcription Regulatory Regions Database (TRRD, http://wwwmgs.bionet.nsc.ru/mgs/gnw/trrd/).

### Statistical analysis

Blood biochemical indexes among the CEN, F1, F1SP, F2 and F2SP groups were analyzed by One-way ANOVA using SPSS software (13.0). When the main effects were significant, means were compared using the Bonferroni post hoc test. Gene expression was analyzed using the DESeq2 package (http://bioconductor.org/packages/release/bioc/html/DESeq2.html), in which the P value was adjusted by the Benjamini-Hochberg (BH) method, belonging to the False Discovery Rate (FDR) for correction. The rest data were analyzed using Graphpad Prism 5.0 (Graphpad Software, San Diego, CA). The associations of blood parameters with age were analyzed using the Pearson relation. Levels of blood parameters between the CEN and the old group (aged 60–80 years) were compared using the Student’s t test. Genetic additive effect (narrow sense) which was measured as the covariance (the slope) of the relationship between centenarians and F1 as well as F1SP was determined according to the method as described^18^. Data are expressed as means ± S.D. and differences of P < 0.05 were considered significant.

## Results

### Blood biochemical indexes

As shown in table 1, there were significant differences in TC (p = 0.005), TG (0.001), LDL-C (p = 0.029), T3 (p < 0.001), FT3 (p < 0.001) and BUA (p = 0.026) among the CEN, F1, F1SP, F2 and F2SP groups. Then we considered the F1SP as the control group and found that TC, LDL-C, TG, T3 and FT3 levels in CEN were significantly lower than that in F1SP (p < 0.05). Of them, LDL-C levels in F1 were lower than the F1SP (p < 0.05). However, we did not observe any significant differences in TC, HDL-C, and TG between the F1 and F1SP groups (p > 0.05).

### Associations of metabolic indexes with age between 20 and 80 years, and between 20 and 100 years

**Figure 1:**
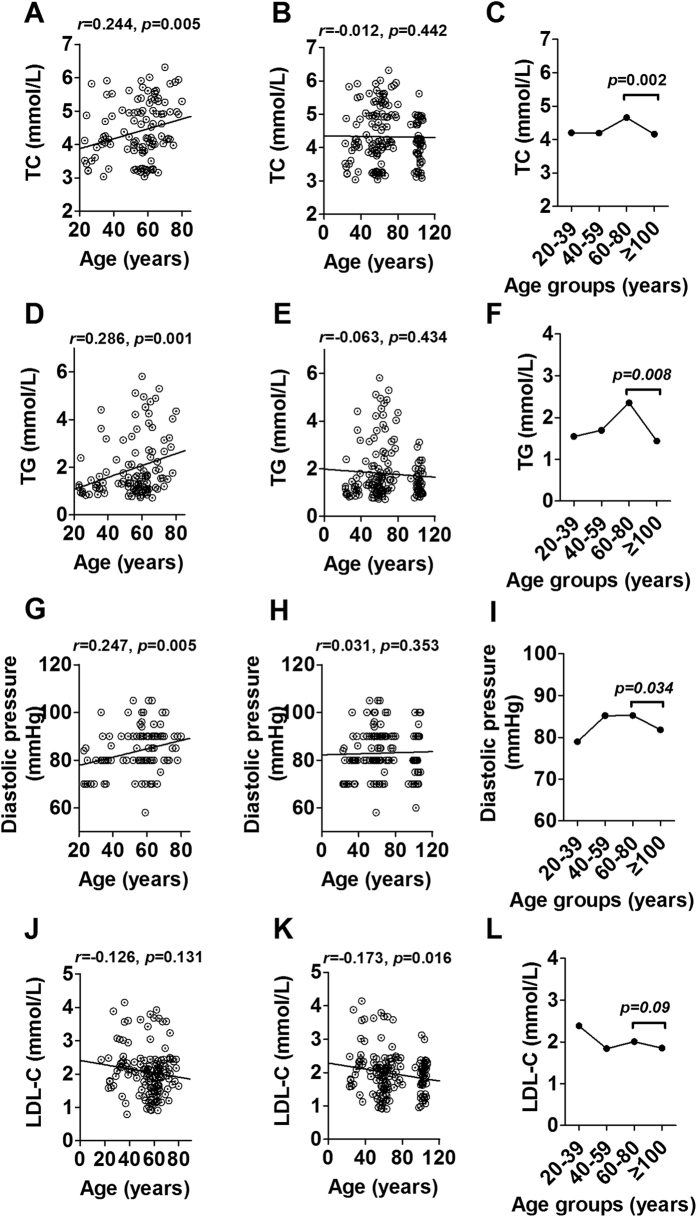
Association of serum total cholesterol (**A,B**), triglyceride (**D,E**), diastolic pressure (**G,H**), LDL-C (**J,K**) with age in subjects aged 20–80 years and 20–110 years from the centenarians’ families, and changes of these parameters in different age groups (**C,F,I,L**). TC, total cholesterol; TG, triglyceride; LDL-C, low density lipoprotein-cholesterol.

To better understand blood index changes in the aging process, we studied their changes with age. The results showed that TC, TG and diastolic pressure were positively associated with age from 20 to 80 years (r = 0.224, p = 0.005; r = 0.286, p = 0.001 and r = 0.247, p = 0.005 respectively, Fig. 1A,D,G). However, the associations were not significant after bringing CEN into analysis (r = −0.012, p = 0.442; r = −0.063, p = 0.434 and r = 0.031, p = 0.353, respectively) (Fig. 1B,E,H), suggesting a changed direction for these parameters in CEN. Then we divided all subjects into four age groups, 20–39 years, 40–59 years, 60–80 years and ≥100 years, and considered the subjects aged 60–80 as the control. As a result, these three parameters were shown significantly lower in CEN than that in the control group (p < 0.05, Fig. 1C,F,I). Different from the change of diastolic pressure, systolic pressure increased dramatically with age from 20 to over 100 years (r = 0.430, p < 0.001 for 20–80 years, supplemental Fig. 1A; r = 0.435, p < 0.001 for 20–100 years, supplemental Fig. 1B), and showed a higher level in CEN than the old subjects (p < 0.05, supplemental Fig. 1C). In addition, we noticed some blood indexes did not change with age between 20 and 80 years, but significantly associated with age between 20 and 100 years when bringing the CEN into analysis, such as LDL-C (r = −0.126, p = 0.131 for 20–80 years, Fig. 1J; r = −0.173, p = 0.016 for 20–100 years, Fig. 1K). After dividing the subjects into four groups, the LDL-C failed to reach significance, but still had a decreased trend (p = 0.09, Fig. 1L). The rest blood indexes were not associated with age (20–80 years) (supplemental Fig. 2), and did not have any changes in CEN (supplemental Fig. 3).

### Maintainance of kidney function parameters in centenarians

**Figure 2:**
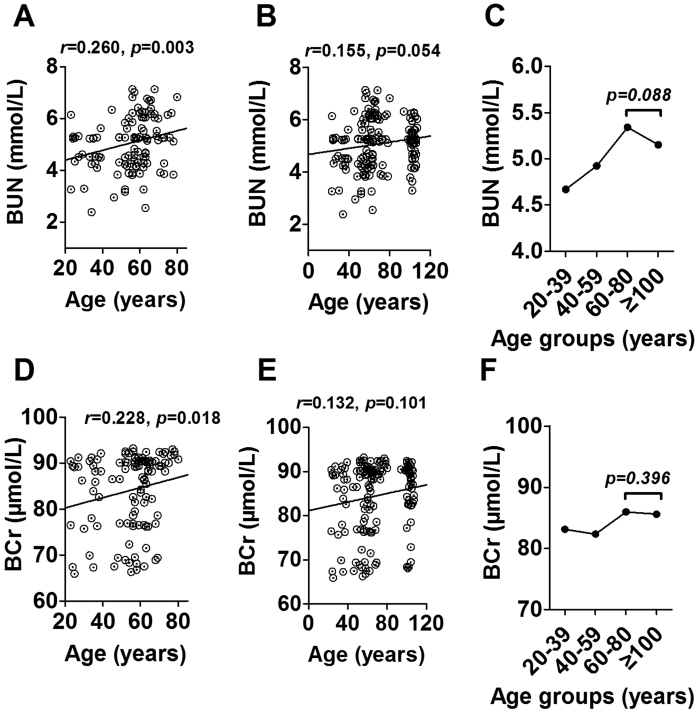
Association of BUN (**A,B**) and BCr (**D,E**) with age in subjects aged 20–80 years and 20–110 years from the centenarians’ families, and changes of these two parameters in different age groups (**C,F**). BUN, blood urea nitrogen; BCr, blood creatinine.

We next evaluated BUA, BUN and BCr levels to reflect the kidney function in the longevity family members. Of them, BUA was not associated with age neither between 20–80 years nor between 20–100 years (supplemental Figs. 2J and 3J). However, BUN and BCr were positively associated with age ranging from 20 to 80 years (r = 0.260, p = 0.003, Fig. 2A; r = 0.228, p = 0.018, Fig. 2D), suggesting a worsening trend for kidney function during the aging process. Similar to the changes in TC, TG and diastolic blood pressure, the associations of BUN and BCr with age were not significant after including CEN (r = 0.155, p = 0.054, Fig. 2B; r = 0.132, p = 0.101, Fig. 2E). Further analysis showed a decreased trend for BUN (p = 0.088, Fig. 2C) and a plateua for BCr (p = 0.396, Fig. 2F) between CEN and the old subjects aged 60–80 years, suggesting the kidney function decreased with age until 80 years and were improved in CEN.

### Associations of blood indexes between centenarians and their offspring

**Figure 3:**
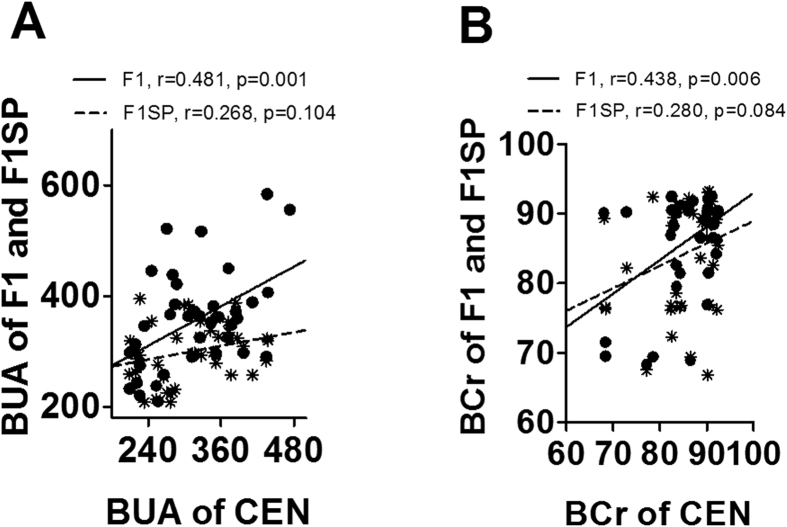
Association of BUA (**A**) and BCr (**B**) levels in CEN with that in F1 and F1SP. BUA, blood urea acid; BCr, blood creatinine; CEN, centenarians; F1, centenarians’ first generation of offspring (F1); F1SP, spouses of F1.

Above association analysis identified blood factors associated with age, especially with the longevity phenotype. Next we performed a genetic analysis (narrow sense) to seek potential heritable parameters between CEN and F1. As a result, BUA and BCr, two important indicators for kidney function, in CEN were significantly and positively related to that in F1SP (r = 0.481, p = 0.001, Fig. 3A and r = 0.438, p = 0.006, Fig. 3B, respectively) but not with F1SP (r = 0.268, p = 0.104, Fig. 3A and r = 0.280, p = 0.084, Fig. 3B, respectively). There were no associations for other parameters between CEN and F1 offspring (supplemental Fig. 4).

### Genes involved in lipid metabolism

**Figure 4:**
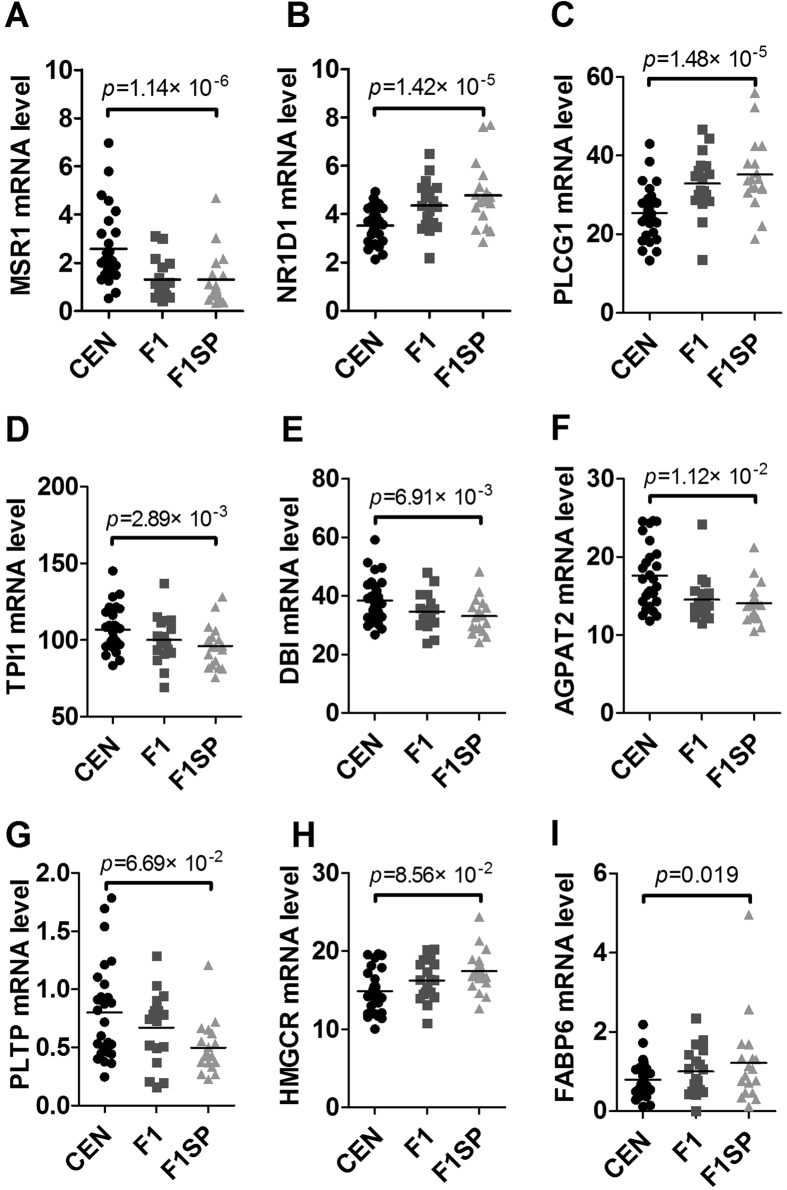
Expression of genes associated with lipid metabolism in the CEN, F1 and F1SP groups. CEN, centenarians; F1, centenarians’ first generation of offspring (F1); F1SP, spouses of F1.

Since there was a significant difference in serum lipids (TC, TG and LDL-C) in CEN compared to the old subjects aged 60–80 years. We wondered how they were regulated. Among the genes involved in lipid metabolism, the *MSR1* (p = 1.14 × 10^−6^), *NR1D1* (p = 1.42 × 10^−5^), *PLCG1* (p = 1.48 × 10^−5^), *TPI1* (p = 2.89 × 10^−3^), *DBI* (p = 6.91 × 10^−3^), *AGPAT2* (p = 1.12 × 10^−2^), *PLTP* (p = 6.69 × 10^−2^), *HMGCR* (p = 8.56 × 10^−2^), *FABP6* (p = 0.019), *DGAT1* (p = 0.028) and *NR1H3* (p = 0.029) in CEN were expressed differently from that in F1SP (Fig. 4A–I and supplemental table 2). After adjustment by the BH method, the *MSR1* (p = 1.05 × 10^−3^), *NR1D1* (p = 7.5 × 10^−3^), *PLCG1* (p = 7.76 × 10^−3^), *TPI1* (p = 6.13 × 10^−3^), *DBI* (p = 0.011), *AGPAT2* (p = 0.015) and *PLTP* (p = 0.049) remained significant, but the *HMGCR* (p = 0.057), *FABP6* (p = 0.095), *DGAT1* (p = 0.119) and *NR1H3* (p = 0.122) were not significant (supplemental table 2). There were not any differences in the expression of these genes between F1 and F1SP except for *SCARB1* (p = 0.01, supplemental table 2), which were not significant after adjustment (p = 0.75, supplemental table 2). Among these genes, the expression of *TPI1* (r = 0.507, p = 0.046, supplemental Fig. 5A) in CEN was shown to associate with that in F1 but not in F1SP (r = −0.166, p = 0.294, supplemental Fig. 5A).

### Genes involved in blood pressure

**Figure 5:**
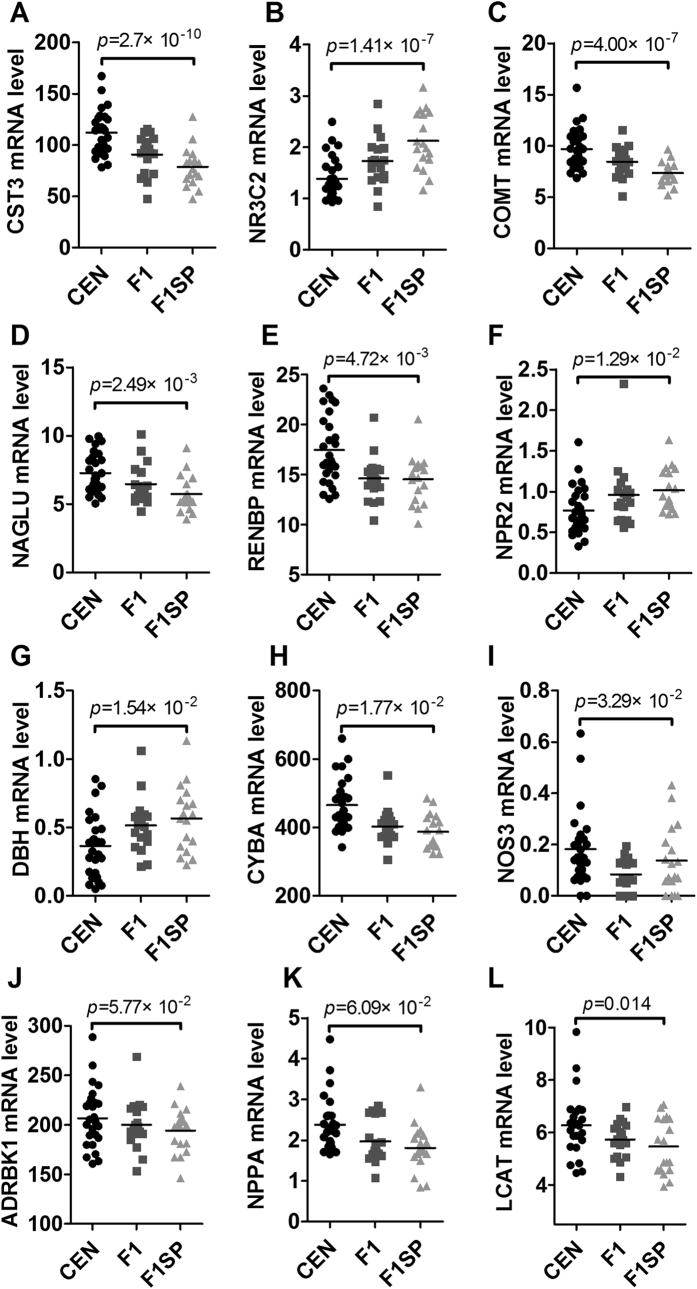
The expression of genes associated with blood pressure in the CEN, F1 and F1SP groups. CEN, centenarians; F1, centenarians’ first generation of offspring (F1); F1SP, spouses of F1.

**Figure 6:**
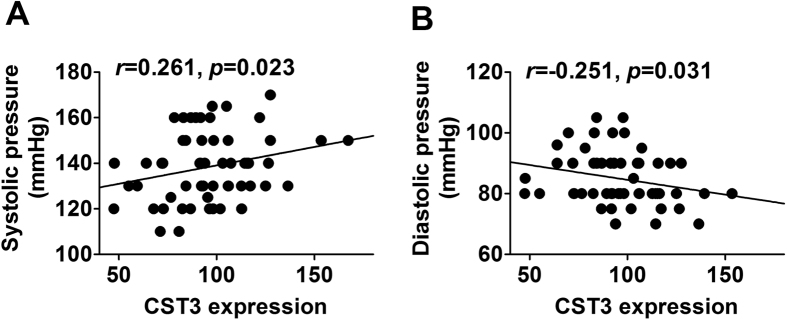
Associations of CST3 expressions with systolic pressure (**A**) and diastolic pressure (**B**). CST3, cystatin (**C**).

Genes associated with blood pressure were listed in supplemental table 3. As shown in Fig. 5 and supplemental 3, there were significant differences in *CST3* (p = 2.7 × 10^−10^), *NR3C2* (p = 1.41 × 10^−7^), *COMT* (p = 4.0 × 10^−7^), *NAGLU* (p = 2.49 × 10^−3^), *RENBP* (p = 4.72 × 10^−3^), *NPR2* (p = 1.29 × 10^−2^), *DBH* (p = 1.54 × 10^−2^), *CYBA* (p = 1.77 × 10^−2^), *NOS3* (p = 3.29 × 10^−2^), *ADRBK1* (p = 5.77 × 10^−2^), *NPPA* (p = 6.09 × 10^−2^), *LCAT* (p = 0.014), *TNFRSF1B* (p = 0.017), *NPR3* (p = 0.019), *NEDD4L* (p = 0.027), *F2R* (p = 0.037) and *GSR* (p = 0.043) between the CEN and F1SP. After adjustment, the *CST3* (p = 1.29 × 10^−7^), *NR3C2* (p = 2.14 × 10^−5^), *COMT* (p = 4.76 × 10^−5^), *NAGLU* (p = 5.61 × 10^−2^), *RENBP* (p = 8.52 × 10^−2^), *NPR2* (p = 0.017), *DBH* (p = 0.019), *CYBA* (p = 0.021), *NOS3* (p = 0.031), *ADRBK1* (p = 0.044) and *NPPA* (p = 0.046) remained significant. However, the rest of above genes were not significant (supplemental table 3). The expression of *NR3C2* and *COMT* in F1 was significantly different from that in F1SP (p = 0.033 and 0.024, respectively), which however, were not significant after the BH adjustment (supplemental table 3). As the most significantly changed gene, *CST3* expression was positively associated with systolic blood pressure (r = 0.261, p = 0.023, Fig. 6A), but negatively with diastolic blood pressure (r = −0.251, p = 0.031, Fig. 6B). The expression of *ADRBK1* (r = 0.552, p = 0.031, supplemental Fig. 5B), *F2R* (r = 0.83, p = 0.0004, supplemental Fig. 5C), *GSR* (r = 0.863, p = 5 × 10^−4^, supplemental Fig. 5D) in CEN was associated with that in F1 but not in F1SP (r = −0.202, p = 0.254, supplemental Fig. 5B; r = −0.359, p = 0.229, supplemental Fig. 5C; r = −0.229, p = 0.225, supplemental Fig. 5D, respectively).

### Genes associated with kidney function

**Figure 7:**
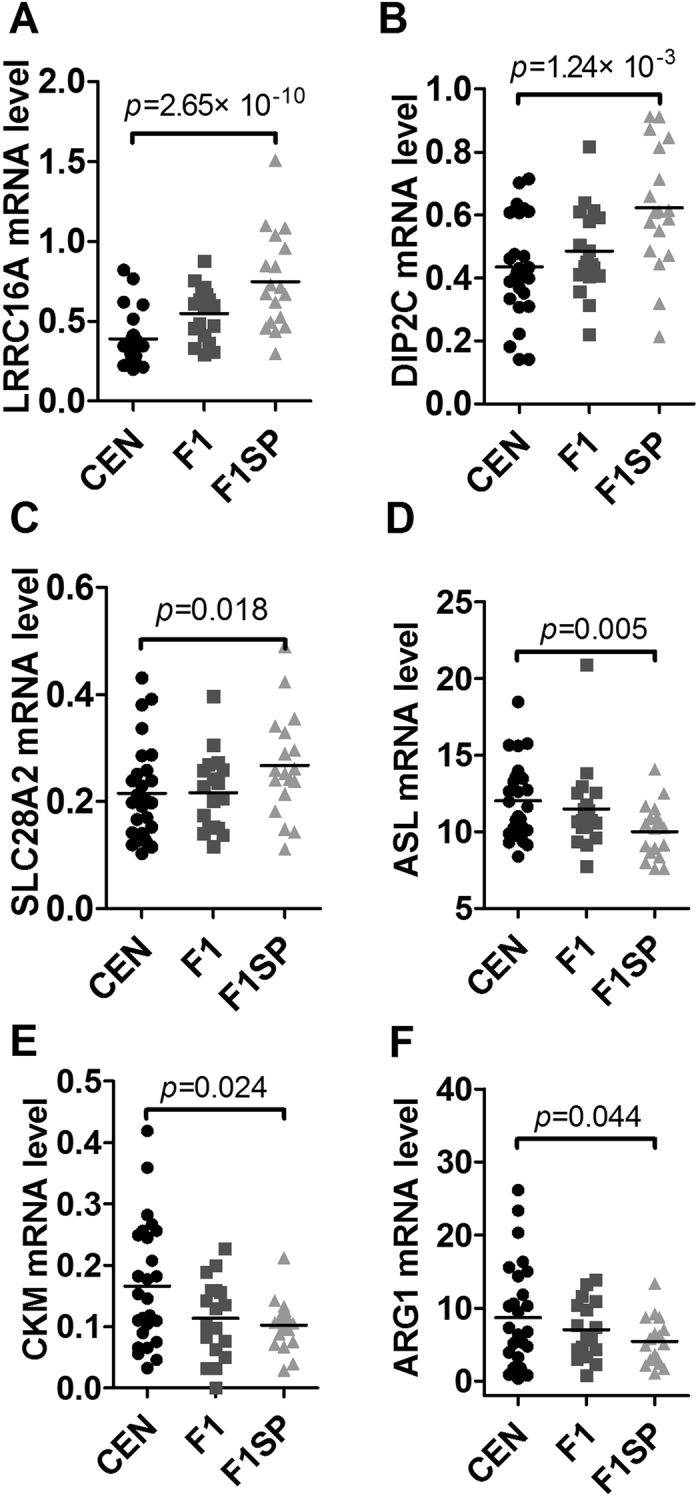
Expression of genes associated with renal function in the CEN, F1 and F1SP groups. CEN, centenarians; F1, centenarians’ first generation of offspring; F1SP, spouses of F1.

Similarly, we collected genes associated with kidney function and investigated their gene expressions. As shown in Fig. 7 and supplemental table 4, we found *LRRC16A* (p = 2.65 × 10^−10^, Fig. 7A), *DIP2C* (p = 1.24 × 10^−3^, Fig. 7B) and *SLC28A2* (p = 0.018, Fig. 7C) were lowly expressed in UA between the CEN versus F1, and *ASL* (p = 0.005, Fig. 7D), *CKM* (p = 0.024, Fig. 7E) and *ARG1* (p = 0.044, Fig. 7F) were highly expressed. Only the expression of *LRRC16A* (p = 0.008), *DIP2C* (0.031) and *ASL* (p = 0.032) was different between the F1 and F1SP, which were not significant after the BH adjustment (supplemental table 4). Among these genes, the expression of *LRRC116A* (r = 0.557, p = 0.047, supplemental Fig. 5G) and *ARG1* (r = 0.558, p = 0.023, supplemental Fig. 5H) in CEN was associated with that in F1 but not in F1SP (r = 0.226, p = 0.19, supplemental Fig. 5E and r = −0.039, p = 0.449, supplemental Fig. 5F, respectively).

## Discussion

Centenarians are an ideal population that is used to study human healthy aging because their lower prevalence or delayed onset of age-related diseases, especially those with high mortality. Identifying any influential factors for these diseases in centenarians may provide insights into helping the old people to improve their healthy status.

In this study, using association and further comparison analyses we identified several blood parameters that may contribute to longevity. First, TC and TG increased with age until 80 years, but decreased in centenarians, indicating that lipid metabolism was improved in the oldest old. A similar trend was observed for LDL-C, although it was not associated with age before 80 years. The changes in lipid levels were consistent with that in other studies^19^,^20^. Increased TC, TG and LDL-C concentrations are the most important independent risk factors for cardiovascular disease^21^, the leading cause of adult death worldwide. In this regard, we can assume that the CEN may be less susceptible to cardiovascular disease, and hence, live longer. To understand why the CEN have such a favorable lipid profile, we analyzed the expression of genes involved in lipid metabolism and found some differentially expressed genes between the CEN and F1SP, such as *MSR1*, *NR1D1*, *PLCG1*, *TPI1*, *DBI*, *AGPAT2*, *PLTP*, *HMGCR* and *FABP6*. Of them, some (*MSR1*, *TPI1*, *DBI*, *AGPAT2* and *PLTP*) were upregulated while some (*NR1D1*, *PLCG1*, *HMGCR* and *FABP6*) were downregulated in CEN versus F1SP. Based on their known functions, they may confer both beneficial and detrimental effect on regulating lipid profiles, suggesting there is a balance in the regulation of the lipid metabolism in the longevity subjects. However, the overall outcome seemed to reduce lipid levels and thus accounted for the favorable lipid profile in centenarians. Of course, there exist many other influential factors that may explain the favorable lipid level in CEN, such as hormone regulation^22^,^23^ and gene polymorphic variants^20^,^24^,^25^,^26^.

More importantly, we observed for the first time that diastolic blood pressure rather than systolic pressure was improved in CEN compared to the elderly. Blood pressure is a well accepted cause for age-related diseases, not only for the cardiovascular disease, but also for cerebrovascular and/or neurodegenerative diseases, such as cerebral hemorrhage and senile dementia^27^. Indeed, a number of studies have noticed that diastolic blood pressure exerts stronger influence than systolic blood pressure on the occurrence and development of cardiovascular and cerebrovascular diseases^28^,^29^. Our results expand the knowledge by extending the age range to over 100 years. Likewise, we managed to identify several candidate genes associated blood pressure. Of notice is the *CST3* gene, it has the most significant difference between the CEN and F1SP, and it codes a protein called cystatin c which has been positively associated with systolic pressure but inversely with diastolic pressure in the 24 h ambulatory blood pressure monitoring^30^, which was well consistent with our observation. However, due to the complex regulation mechanisms on blood pressure, it is hard to distinguish all significant genes involved in diastolic blood pressure from those in systolic blood pressure. In addition, we found BUN and Bcr, two important indexes for kidney function, increased with age (20–80 years), indicating that the kidney function worsens with aging, being consistent with results got in other studies^31^,^32^. Surprisingly, they did not increase with age but were maintained on a plateau in CEN, suggesting that the worsening kidney function slowed down in the longevity subjects. Accordingly, genes such as the *LRRC16A*, *DIP2C* and *SLC28A2*, were differentially expressed between CEN and F1SP, being likely involved in the maintenance of kidney function^33^,^34^, however, the causal relationship and mechanisms are still unclear. The kidneys play important roles in the removal of metabolic waste products and maintenance of water-electrolyte balance and blood pressure, and therefore renal function is essential for homeostasis. Diseased kidney function is frequently associated with cardiovascular disease (CVD)^35^,^36^. Patients with chronic kidney disease also have a high prevalence of arteriosclerosis^36^. Therefore, the maintenance of kidney function may associate with the lower incidence of CVD in CEN.

Longevity is an extreme phenotype of aging. CEN manage to live to or over 100 years of age, their descendants are prone to achieve longevity, suggesting the possible involvement of genetic factors. In our previous studies, we identified some protective or risk mutations in CEN^14^,^15^, mainly associated with insulin pathway and vascular diseases^14^,^15^, respectively. In this study, BUA and BCr in CEN were identified to be two potential heritable indexes due to their significant associations between the CEN and F1 rather than the F1SP. This result provides additional informative explanation for the lower incidence of some fatal diseases, e.g. cardiovascular diseases^4^, in CEN and their descendants. Accordingly, the expression of *LRRC16A* and *ARG1* in CEN were highly associated with that in F1, but not F1SP, being the candidate genes for the inheritance.

In summary, our results revealed that the improvements of serum lipids, kidney function, and especially the diastolic blood pressure but not systolic blood pressure as beneficial factors for longevity. Of them, improved kidney function may be a heritable phenotype that could explain the lower prevalence of age-related diseases to some extent in both CEN and their descendants.

## Additional Information

**How to cite this article**: He, Y.-H. *et al.* Improved lipids, diastolic pressure and kidney function are potential contributors to familial longevity: a study on 60 Chinese centenarian families. *Sci. Rep.* **6**, 21962; doi: 10.1038/srep21962 (2016).

## Supplementary Material

Supplementary Information

## Figures and Tables

**Table 1:** Blood biochemical indexes in centenarian families.

	CEN (n = 61)	F1 (n = 63)	F1SP (n = 47)	F2 (n = 25)	F2SP (n = 10)
TC (mmol/L)	4.13** **±** **0.68	4.50** **±** **0.79*	4.43** **±** **0.93*	4.48** **±** **0.91	3.72** **±** **0.42#
HDL-C (mmol/L)	1.83** **±** **0.40	1.79** **±** **0.16	1.82** **±** **0.14	1.85** **±** **0.12	1.70** **±** **0.16
LDL-C (mmol/L)	1.88** **±** **0.59	1.84** **±** **0.41#	1.97** **±** **0.76*	2.36** **±** **0.87*	1.94** **±** **0.47
TG (mmol/L)	1.47** **±** **0.58	2.06** **±** **1.24*	1.95** **±** **1.19*	2.03** **±** **1.30	1.01** **±** **0.17
Glucose (mmol/L)	6.03** **±** **1.33	6.34** **±** **1.24	5.87** **±** **0.83	6.33** **±** **1.35	5.97** **±** **0.93
TSH (IU/mL)	1.80** **±** **1.23	1.39** **±** **1.01	1.43** **±** **0.85	1.2** **±** **0.75	1.45** **±** **0.84
T3 (nmol/L)	1.29** **±** **0.22	1.52** **±** **0.25*	1.52** **±** **0.31*	1.8** **±** **1.10*	1.37** **±** **0.14
T4 (nmol/L)	103.66** **±** **17.37	106.95** **±** **20.74	108.11** **±** **20.05	110.22** **±** **42.41	100.15** **±** **15.31
FT3 (pmol/L)	3.68** **±** **0.64	4.67** **±** **0.76*	4.49** **±** **0.80*	6.04** **±** **4.15*	4.38** **±** **0.60
FT4 (pmol/L)	16.15** **±** **2.93	16.55** **±** **2.34	15.74** **±** **2.72	18.95** **±** **10.06*	16.63** **±** **4.68
ALT (U/L)	27.31** **±** **5.96	29.63** **±** **6.67	28.47** **±** **6.69	30.27** **±** **7.39	28.79** **±** **6.21
AST (U/L)	24.96** **±** **4.88	26.57** **±** **6.59	25.61** **±** **6.62	27.30** **±** **7.61	25.28** **±** **6.09
Total protein (g/L)	65.61** **±** **2.36	65.93** **±** **2.63	65.96** **±** **2.91	66.17** **±** **2.41	65.58** **±** **1.59
Albumin (g/L)	39.12** **±** **1.14	39.29** **±** **1.69	39.38** **±** **2.27	39.40** **±** **1.15	38.74** **±** **0.97
Globlin (g/L)	26.5** **±** **1.87	26.64** **±** **1.99	26.37** **±** **2.15	26.76v±** **1.96	26.84** **±v2.00
Ratio of albumin to globlin	1.48** **±** **0.11	1.69** **±** **1.63	1.51** **±** **0.14	1.48** **±** **0.11	1.44** **±** **0.13
Total bilirubin (μmol/L)	11.3** **±** **1.30	11.34** **±** **1.59	11.41** **±** **1.76	11.02** **±** **1.40	11.32** **±** **1.32
Direct bilirubin (μmol/L)	2.57** **±** **0.55	2.44v±** **0.55	2.49** **±** **0.68	2.76** **±v0.46	2.64v±** **0.30
Indirect bilirubin (μmol/L)	8.72** **±v1.29	8.89** **±** **1.49	8.81** **±** **1.74	7.98** **±** **1.97	8.68** **±** **1.41
BUA (μmol/L)	316.7** **±** **70.92	344.81** **±** **87.27#	301.02** **±** **52.44	342.36** **±** **106.98	328.75** **±** **64.06
BCr (μmol/L)	85.34** **±** **7.25	85.33** **±** **7.90	83.92** **±** **7.95	85.13** **±** **7.64	83.38** **±** **9.48
BUN (μmol/L)	5.16** **±** **0.75	5.13** **±** **0.95	5.07** **±** **1.25	4.98** **±** **0.83	4.48** **±** **0.83

^*^p < 0.05 versus CEN group.

^#^p < 0.05 versus F1SP group; CEN, centenarians; F1, first generation of offspring; F1SP, F1 spouses; F2, second generation of offspring; F2SP, F2 spouses; TC, total cholesterol; HDL-C, high density lipoprotein-cholesterol; LDL-C, low density lipoprotein-cholesterol; TG, triglyceride; TSH, thyroid-stimulating hormone; T3, triiodothyronine; T4, thyroxine; FT3, free triiodothyronine; FT4, free thyroxine; ALT, alanine aminotransferase; AST, aspartate aminotransferase; BUA, blood uric acid; BCr, blood creatinine.
