# Supplementary material for: Improved lipids, diastolic pressure and kidney function are potential contributors to familial longevity: a study on 60 Chinese centenarian families
Source: Sci Rep. 2016 Feb 25;6:21962. doi: 10.1038/srep21962 (PMC4766395; doi:10.1038/srep21962)
Supplement: Supplementary Information [file srep21962-s1.doc]

**Supplemental materials**

**Improved** **lipids, diastolic pressure and kidney function are potential** **contributors to familial longevity: a study on 60 Chinese centenarian families**

Yong-Han He, Shao-Yan Pu, Fu-Hui Xiao, Xiao-Qiong Chen, Dong-Jing Yan, Yao-Wen Liu, Rong Lin, Xiao-Ping Liao, Qin Yu, Li-Qin Yang, Xing-Li Yang, Ming-Xia Ge, Ying Li, Jian-Jun Jiang, Wang-Wei Cai, Qing-Peng Kong

Supplemental figure 1.


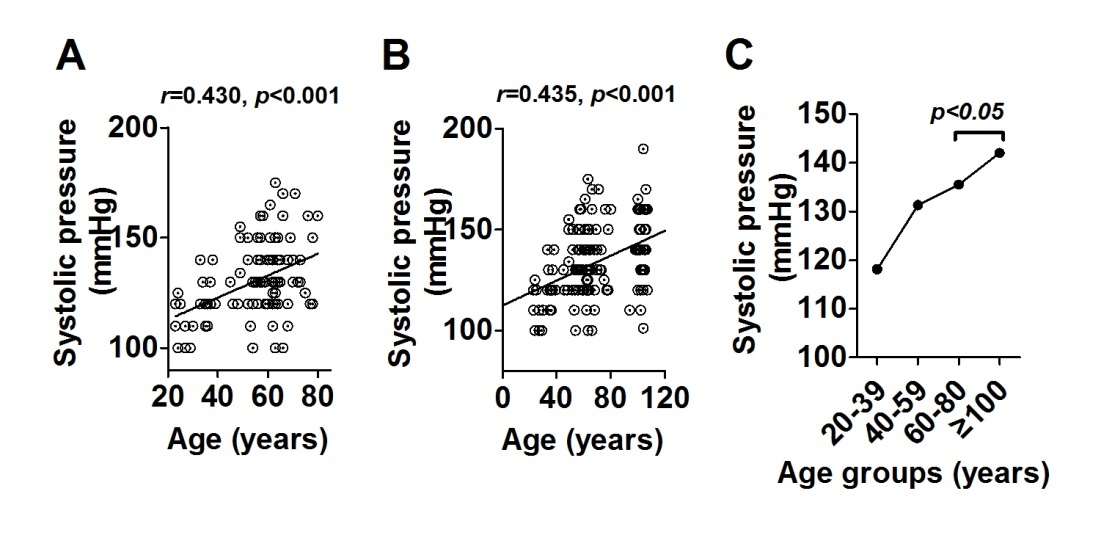


**Supplemental figure 1.** Association of systolic pressure with age in subjects aged 20-80 years (A) and 20-110 years (B) from the centenarians’ families, and changes of systolic pressure in different age groups (C).

Supplemental figure 2.


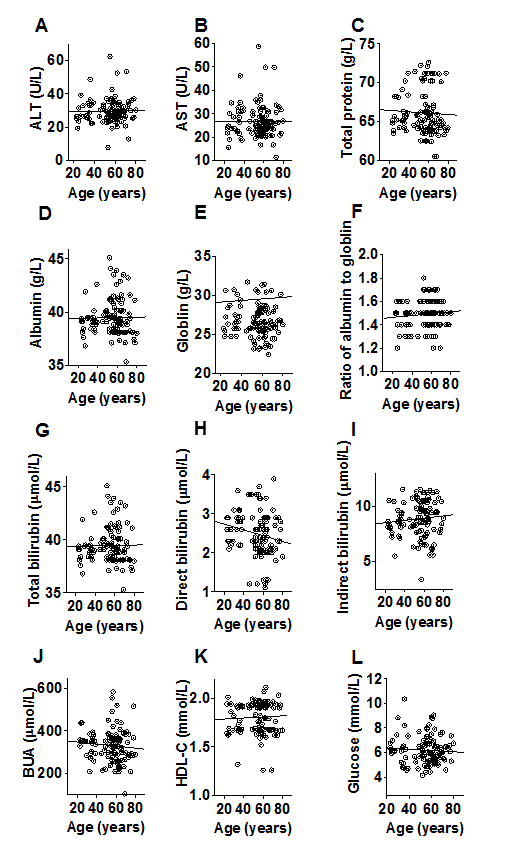


**Supplemental figure 2.** Association of blood biochemical indexes with age in subjects aged 20-80 years. ALT, alanine aminotransferase; AST, aspartate aminotransferase; BUA, blood urea acid; HDL-C, high density lipoprotein-cholesterol.

Supplemental figure 3.


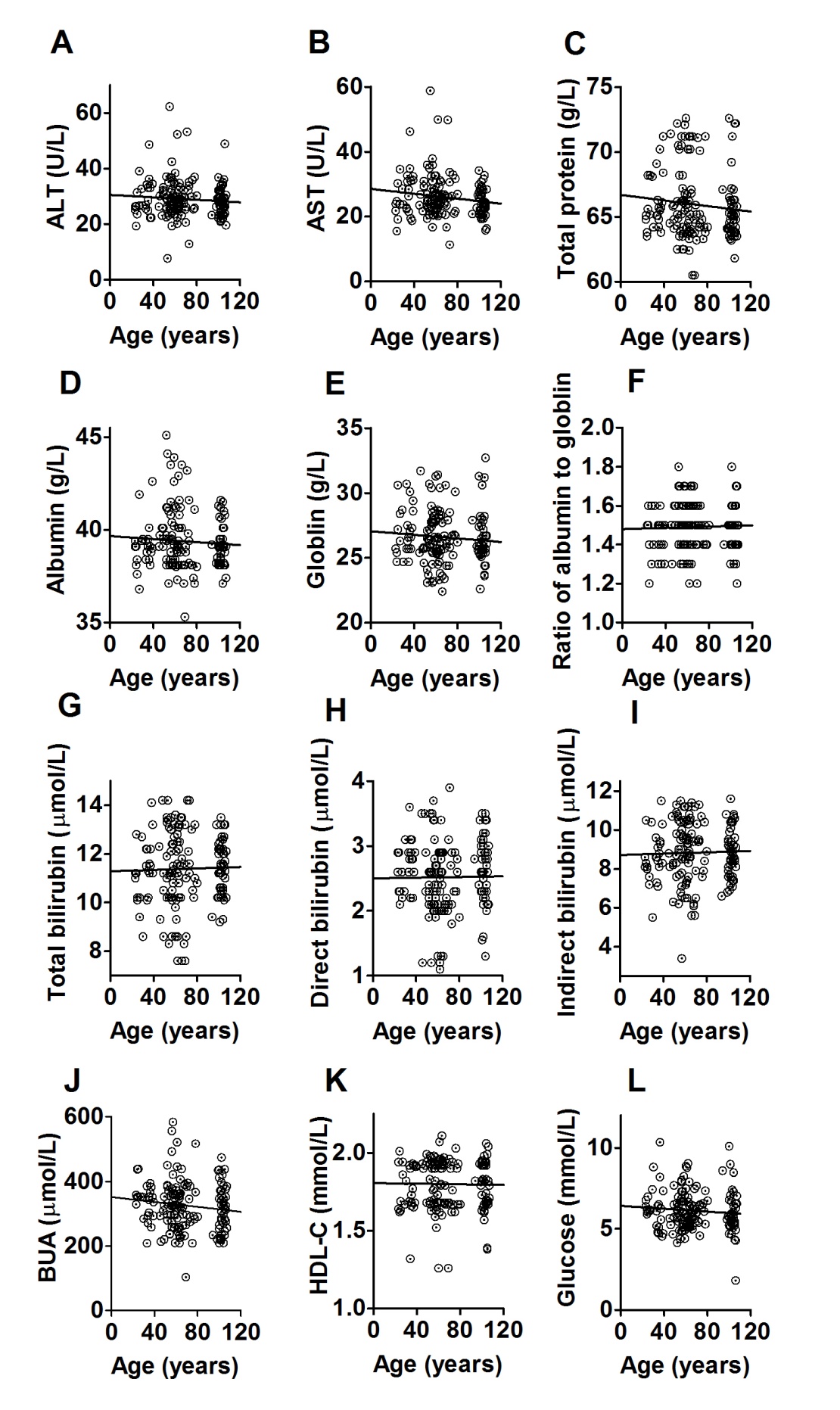


**Supplemental figure 3.** Association of blood biochemical indexes with age in subjects aged 20-100 years. ALT, alanine aminotransferase; AST, aspartate aminotransferase; BUA, blood urea acid; HDL-C, high density lipoprotein-cholesterol.

Supplemental figure 4.


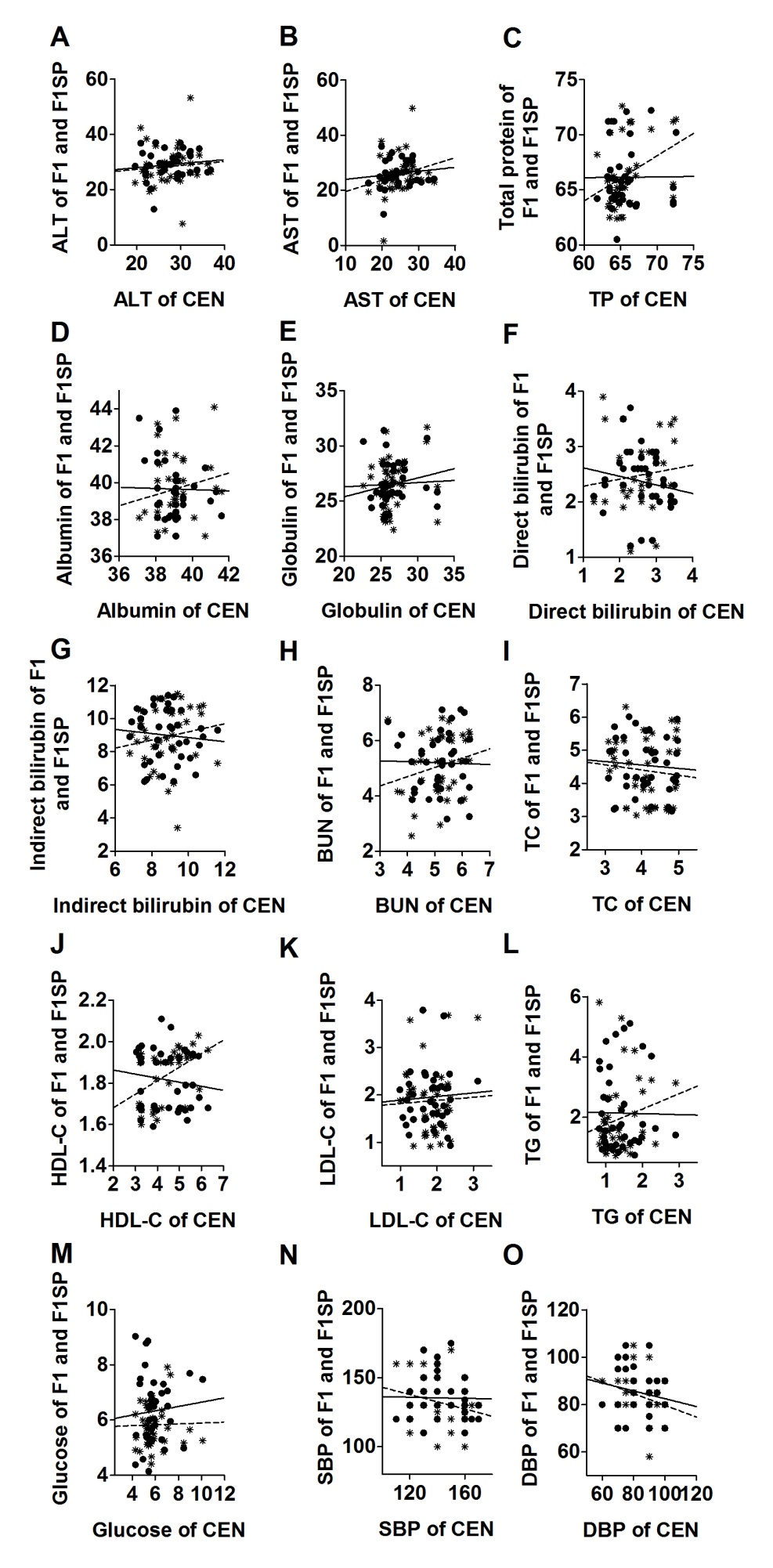


**Supplemental figure 4.** Association of blood biochemical indexes in CEN with that in F1 and F1SP. ALT, alanine aminotransferase; AST, aspartate aminotransferase; BUA, blood urea acid; HDL-C, high density lipoprotein-cholesterol; CEN, centenarians; F1, first generation of offspring (F1); F1SP, spouses of F1.

Supplemental figure 5.


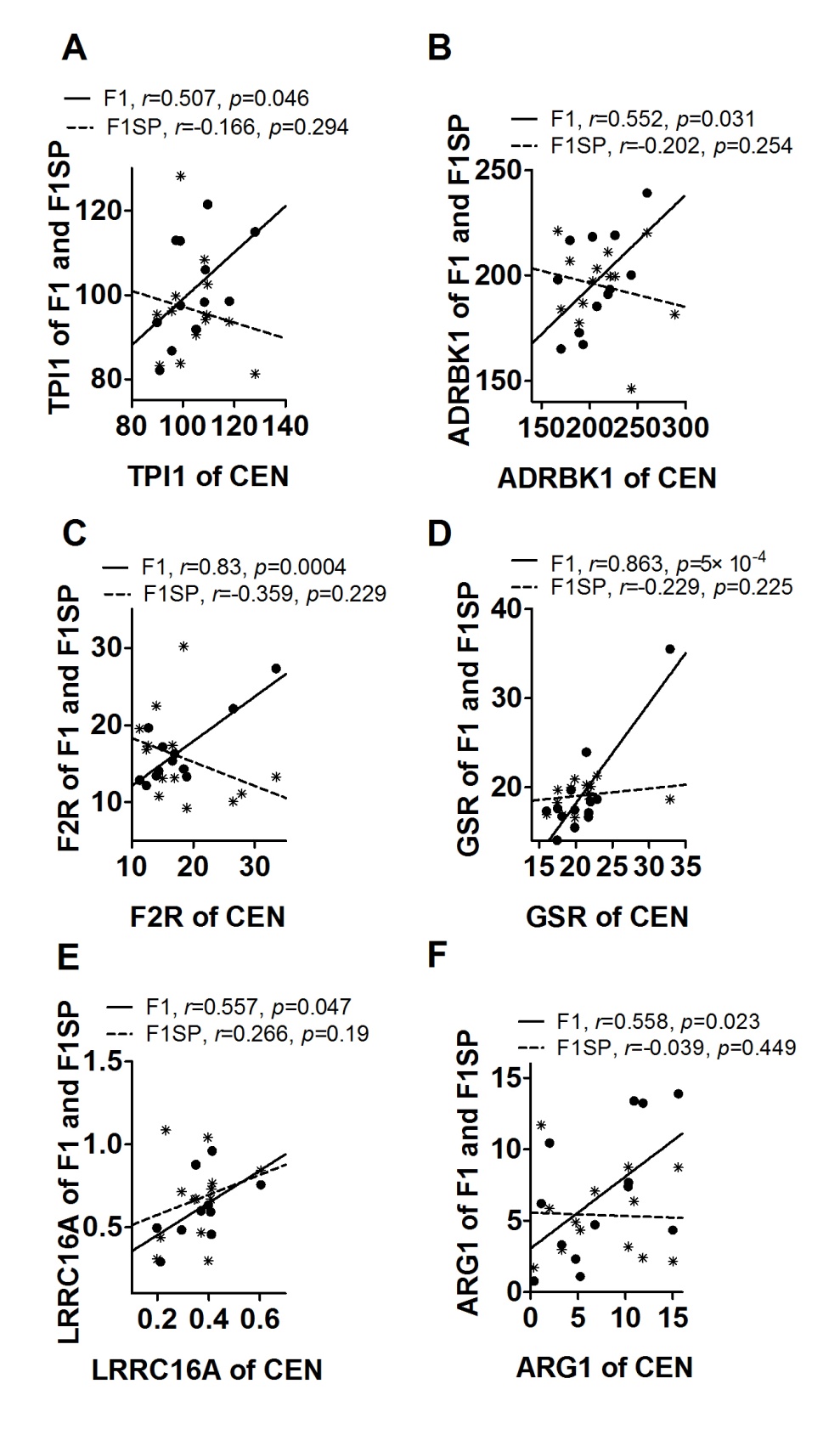


**Supplemental figure 5.** Association of gene expression in CEN with that in F1 and F1SP. CEN, centenarians; F1, centenarians’ first generation of offspring (F1); F1SP, spouses of F1.

| **Supplemental table 1. Samples for gene expression association analysis.** | | | |
| --- | --- | --- | --- |
| **Family** | **CEN ID** | **F1 ID** | **F1SP ID** |
| 1 | 1 | 3 | 17 |
| 2 | 85 | 86 | 89 |
| 3 | 90 | 91 | 92 |
| 4 | 101 | 102 | 103 |
| 5 | 110 | 111 | 112 |
| 6 | 119 | 120 | 121 |
| 7 | 122 | 124 | 123 |
| 8 | 141 | 142 | 143 |
| 9 | 164 | 160 | 161 |
| 10 | 169 | 170 | 172 |
| 11 | 184 | 185 | 186 |
| 12 | 203 | 204 | 205 |
| 13 | 231 | 232 | 233 |
| CEN, centenarians; F1, first generation of offpring; F1SP; spouses of F1 | | | |

| **Supplemental table 2. Expression comparisons of genes associated with lipid metabolism.** | | | | | | | | | |
| --- | --- | --- | --- | --- | --- | --- | --- | --- | --- |
|  |  | **CEN vs. F1SP** |  |  |  |  | **F1 vs. F1SP** |  |  |
| **Gene name** | **Base Mean** | **log2FoldChange** | **p** | **adjusted p** | | **Base Mean** | **log2FoldChange** | **p** | **adjusted p** |
| MSR1* | 173.964 | 0.696 | 0.000 | 0.000 |  | 173.964 | 0.114 | 0.432 | 1.000 |
| NR1D1* | 203.351 | -0.412 | 0.000 | 0.001 |  | 203.351 | -0.113 | 0.266 | 1.000 |
| PLCG1* | 2712.738 | -0.378 | 0.000 | 0.001 |  | 2712.738 | -0.087 | 0.354 | 1.000 |
| TPI1* | 3331.295 | 0.243 | 0.000 | 0.006 |  | 3331.295 | 0.055 | 0.450 | 1.000 |
| DBI* | 909.624 | 0.260 | 0.001 | 0.011 |  | 909.624 | 0.049 | 0.552 | 1.000 |
| AGPAT2* | 439.016 | 0.306 | 0.001 | 0.015 |  | 439.016 | 0.063 | 0.535 | 1.000 |
| PLTP* | 26.480 | 0.371 | 0.007 | 0.049 |  | 26.480 | 0.247 | 0.080 | 1.000 |
| HMGCR* | 1311.263 | -0.209 | 0.009 | 0.057 |  | 1311.263 | -0.094 | 0.272 | 1.000 |
| FABP6* | 15.059 | -0.340 | 0.019 | 0.095 |  | 15.059 | -0.135 | 0.349 | 1.000 |
| DGAT1* | 1285.025 | 0.165 | 0.028 | 0.119 |  | 1285.025 | 0.098 | 0.226 | 1.000 |
| NR1H3* | 79.102 | 0.218 | 0.029 | 0.122 |  | 79.102 | -0.157 | 0.144 | 1.000 |
| PPARG | 8.993 | 0.258 | 0.075 | 0.219 |  | 8.993 | 0.138 | 0.343 | 1.000 |
| FDFT1 | 1351.842 | 0.089 | 0.084 | 0.235 |  | 1351.842 | 0.043 | 0.446 | 1.000 |
| LRP1 | 5782.635 | 0.189 | 0.113 | 0.284 |  | 5782.635 | 0.055 | 0.660 | 1.000 |
| RARB | 7.182 | -0.221 | 0.127 | 0.304 |  | 7.182 | -0.247 | 0.089 | 1.000 |
| CEBPA | 458.411 | 0.164 | 0.128 | 0.305 |  | 458.411 | 0.088 | 0.439 | 1.000 |
| SCARB1 | 156.181 | 0.135 | 0.184 | 0.378 |  | 156.181 | 0.279 | 0.010 | 0.754 |
| LPL | 21.845 | -0.177 | 0.186 | 0.381 |  | 21.845 | 0.050 | 0.697 | 1.000 |
| CYP27B1 | 5.297 | -0.186 | 0.188 | 0.383 |  | 5.297 | -0.098 | 0.496 | 1.000 |
| FASN | 940.585 | 0.103 | 0.190 | 0.386 |  | 940.585 | 0.122 | 0.147 | 1.000 |
| APOA1 | 3.741 | -0.161 | 0.266 | 0.477 |  | 3.741 | 0.134 | 0.358 | 1.000 |
| APP | 1709.162 | 0.085 | 0.330 | 0.542 |  | 1709.162 | -0.108 | 0.251 | 1.000 |
| SREBF2 | 1791.551 | 0.060 | 0.351 | 0.562 |  | 1791.551 | 0.043 | 0.537 | 1.000 |
| PLA2G2D | 4.639 | -0.106 | 0.465 | 0.660 |  | 4.639 | -0.109 | 0.450 | 1.000 |
| ACOX1 | 2518.744 | -0.084 | 0.507 | 0.694 |  | 2518.744 | 0.124 | 0.350 | 1.000 |
| GHRL | 92.729 | 0.078 | 0.509 | 0.695 |  | 92.729 | -0.053 | 0.670 | 1.000 |
| GHRL | 92.729 | 0.078 | 0.509 | 0.695 |  | 92.729 | -0.053 | 0.670 | 1.000 |
| ABCA1 | 1487.605 | 0.090 | 0.513 | 0.699 |  | 1487.605 | 0.113 | 0.423 | 1.000 |
| LDLR | 543.633 | 0.067 | 0.534 | 0.716 |  | 543.633 | -0.006 | 0.960 | 1.000 |
| CYP7A1 | 1.795 | -0.080 | 0.562 | 0.737 |  | 1.795 | -0.153 | 0.253 | 1.000 |
| ABCG1 | 767.642 | 0.054 | 0.660 | 0.807 |  | 767.642 | 0.096 | 0.455 | 1.000 |
| LIPC | 18.092 | -0.058 | 0.674 | 0.816 |  | 18.092 | 0.060 | 0.654 | 1.000 |
| ACADM | 397.380 | -0.018 | 0.754 | 0.869 |  | 397.380 | -0.015 | 0.812 | 1.000 |
| CPT2 | 190.059 | 0.020 | 0.817 | 0.908 |  | 190.059 | -0.038 | 0.681 | 1.000 |
| ACLY | 2257.772 | 0.008 | 0.862 | 0.933 |  | 2257.772 | 0.006 | 0.903 | 1.000 |
| RARA | 4916.673 | -0.010 | 0.920 | 0.963 |  | 4916.673 | -0.081 | 0.443 | 1.000 |
| APOA2 | 25.877 | 0.002 | 0.991 | 0.996 |  | 25.877 | 0.156 | 0.282 | 1.000 |
| CEN, centenarians; F1, first generation of offspring; F1SP; spouses of F1; *p value of CEN vs. F1SP <0.05 | | | | | | | |  |  |

| **Supplemental table 3. Expression comparisons of genes associated with blood pressure.** | | | | | | | | | | | | | |
| --- | --- | --- | --- | --- | --- | --- | --- | --- | --- | --- | --- | --- | --- |
|  |  | **CEN vs. F1SP** | |  |  |  | | **F1 vs. F1SP** | |  | |  | |
| **Gene name** | **Base Mean** | **log2FoldChange** | **p** | **adjusted p** |  | **Base Mean** | **log2FoldChange** | | **p** | | **adjusted p** | |  |
| CST3* | 4191.475 | 0.553 | 0.000 | 0.000 |  | 4191.475 | 0.169 | | 0.072 | | 0.983 | |  |
| NR3C2* | 178.962 | -0.569 | 0.000 | 0.000 |  | 178.962 | -0.244 | | 0.033 | | 0.923 | |  |
| COMT* | 394.360 | 0.373 | 0.000 | 0.000 |  | 394.360 | 0.180 | | 0.024 | | 0.873 | |  |
| NAGLU* | 326.461 | 0.302 | 0.000 | 0.006 |  | 326.461 | 0.149 | | 0.093 | | 1.000 | |  |
| RENBP* | 416.330 | 0.268 | 0.000 | 0.009 |  | 416.330 | 0.013 | | 0.873 | | 1.000 | |  |
| NPR2* | 54.742 | -0.380 | 0.001 | 0.017 |  | 54.742 | -0.068 | | 0.584 | | 1.000 | |  |
| DBH* | 23.121 | -0.448 | 0.002 | 0.019 |  | 23.121 | -0.088 | | 0.543 | | 1.000 | |  |
| CYBA* | 5502.564 | 0.280 | 0.002 | 0.021 |  | 5502.564 | 0.039 | | 0.683 | | 1.000 | |  |
| NOS3* | 23.564 | -0.373 | 0.003 | 0.031 |  | 23.564 | -0.193 | | 0.144 | | 1.000 | |  |
| ADRBK1* | 12168.475 | 0.160 | 0.006 | 0.044 |  | 12168.475 | 0.027 | | 0.663 | | 1.000 | |  |
| NPPA* | 31.830 | 0.282 | 0.006 | 0.046 |  | 31.830 | 0.085 | | 0.442 | | 1.000 | |  |
| LCAT* | 142.050 | 0.171 | 0.014 | 0.080 |  | 142.050 | 0.058 | | 0.445 | | 1.000 | |  |
| TNFRSF1B* | 14609.867 | 0.227 | 0.017 | 0.087 |  | 14609.867 | 0.106 | | 0.299 | | 1.000 | |  |
| NPR3* | 5.024 | -0.336 | 0.019 | 0.095 |  | 5.024 | -0.068 | | 0.639 | | 1.000 | |  |
| NEDD4L* | 55.396 | -0.234 | 0.027 | 0.118 |  | 55.396 | 0.056 | | 0.617 | | 1.000 | |  |
| F2R* | 1145.794 | 0.220 | 0.037 | 0.143 |  | 1145.794 | 0.061 | | 0.588 | | 1.000 | |  |
| GSR* | 1133.594 | 0.167 | 0.043 | 0.156 |  | 1133.594 | 0.156 | | 0.080 | | 1.000 | |  |
| HP | 108.065 | 0.270 | 0.058 | 0.188 |  | 108.065 | 0.009 | | 0.948 | | 1.000 | |  |
| GNB3 | 8.838 | -0.253 | 0.062 | 0.195 |  | 8.838 | 0.120 | | 0.388 | | 1.000 | |  |
| GNAI1 | 13.262 | -0.249 | 0.069 | 0.210 |  | 13.262 | -0.203 | | 0.149 | | 1.000 | |  |
| PRCP | 1964.304 | 0.124 | 0.092 | 0.248 |  | 1964.304 | -0.067 | | 0.396 | | 1.000 | |  |
| INSR | 222.281 | 0.194 | 0.102 | 0.265 |  | 222.281 | 0.222 | | 0.075 | | 0.983 | |  |
| CAST | 3788.715 | 0.118 | 0.114 | 0.285 |  | 3788.715 | 0.074 | | 0.359 | | 1.000 | |  |
| NR3C1 | 3092.877 | -0.114 | 0.117 | 0.290 |  | 3092.877 | -0.047 | | 0.552 | | 1.000 | |  |
| GNAS | 11255.355 | 0.119 | 0.117 | 0.290 |  | 11255.355 | -0.038 | | 0.645 | | 1.000 | |  |
| PLA2G7 | 144.267 | 0.194 | 0.117 | 0.290 |  | 144.267 | 0.086 | | 0.511 | | 1.000 | |  |
| ADORA2A | 21.091 | -0.208 | 0.118 | 0.291 |  | 21.091 | -0.114 | | 0.408 | | 1.000 | |  |
| ADRB2 | 563.838 | 0.161 | 0.146 | 0.330 |  | 563.838 | 0.041 | | 0.725 | | 1.000 | |  |
| PNMT | 7.266 | 0.210 | 0.147 | 0.331 |  | 7.266 | 0.259 | | 0.075 | | 0.983 | |  |
| KLKB1 | 31.877 | -0.184 | 0.161 | 0.350 |  | 31.877 | 0.114 | | 0.403 | | 1.000 | |  |
| LPL | 21.845 | -0.177 | 0.186 | 0.381 |  | 21.845 | 0.050 | | 0.697 | | 1.000 | |  |
| PLAT | 10.804 | -0.179 | 0.192 | 0.389 |  | 10.804 | -0.029 | | 0.836 | | 1.000 | |  |
| ACSM3 | 40.053 | -0.161 | 0.217 | 0.420 |  | 40.053 | -0.146 | | 0.283 | | 1.000 | |  |
| SLC5A6 | 393.830 | -0.061 | 0.235 | 0.441 |  | 393.830 | 0.019 | | 0.728 | | 1.000 | |  |
| ACE | 83.264 | -0.149 | 0.246 | 0.455 |  | 83.264 | -0.139 | | 0.299 | | 1.000 | |  |
| WNK1 | 6624.634 | -0.107 | 0.251 | 0.460 |  | 6624.634 | 0.014 | | 0.888 | | 1.000 | |  |
| KRIT1 | 781.046 | -0.067 | 0.258 | 0.467 |  | 781.046 | 0.001 | | 0.990 | | 1.000 | |  |
| APOA1 | 3.741 | -0.161 | 0.266 | 0.477 |  | 3.741 | 0.134 | | 0.358 | | 1.000 | |  |
| NISCH | 3067.316 | -0.052 | 0.267 | 0.477 |  | 3067.316 | 0.036 | | 0.479 | | 1.000 | |  |
| SLC8A1 | 942.173 | -0.154 | 0.271 | 0.481 |  | 942.173 | 0.062 | | 0.667 | | 1.000 | |  |
| GRK4 | 12.702 | 0.136 | 0.289 | 0.501 |  | 12.702 | 0.184 | | 0.167 | | 1.000 | |  |
| ADM | 1735.762 | -0.142 | 0.313 | 0.526 |  | 1735.762 | -0.139 | | 0.332 | | 1.000 | |  |
| ADD1 | 4563.680 | 0.036 | 0.325 | 0.538 |  | 4563.680 | 0.034 | | 0.401 | | 1.000 | |  |
| LEPR | 159.196 | -0.095 | 0.357 | 0.568 |  | 159.196 | -0.184 | | 0.094 | | 1.000 | |  |
| GOSR2 | 636.616 | -0.052 | 0.361 | 0.571 |  | 636.616 | 0.013 | | 0.830 | | 1.000 | |  |
| LPXN | 1562.598 | 0.051 | 0.377 | 0.587 |  | 1562.598 | 0.072 | | 0.253 | | 1.000 | |  |
| SGK1 | 7927.615 | 0.127 | 0.379 | 0.588 |  | 7927.615 | 0.119 | | 0.412 | | 1.000 | |  |
| ADRB1 | 11.349 | 0.126 | 0.385 | 0.594 |  | 11.349 | -0.092 | | 0.527 | | 1.000 | |  |
| PF4 | 1720.239 | 0.119 | 0.391 | 0.599 |  | 1720.239 | -0.280 | | 0.049 | | 0.935 | |  |
| MME | 6276.867 | -0.120 | 0.400 | 0.608 |  | 6276.867 | 0.091 | | 0.528 | | 1.000 | |  |
| SOD1 | 1093.186 | -0.074 | 0.403 | 0.610 |  | 1093.186 | -0.110 | | 0.249 | | 1.000 | |  |
| PPBP | 3814.432 | 0.118 | 0.410 | 0.615 |  | 3814.432 | -0.318 | | 0.028 | | 0.885 | |  |
| ECE1 | 4315.455 | -0.096 | 0.433 | 0.635 |  | 4315.455 | 0.018 | | 0.890 | | 1.000 | |  |
| SLC9A1 | 988.588 | 0.054 | 0.440 | 0.641 |  | 988.588 | -0.010 | | 0.894 | | 1.000 | |  |
| SELP | 228.130 | 0.098 | 0.448 | 0.648 |  | 228.130 | -0.262 | | 0.052 | | 0.949 | |  |
| BAIAP2L1 | 61.133 | -0.079 | 0.502 | 0.691 |  | 61.133 | -0.008 | | 0.950 | | 1.000 | |  |
| SLC12A1 | 176.940 | -0.067 | 0.517 | 0.702 |  | 176.940 | -0.060 | | 0.531 | | 1.000 | |  |
| THBD | 1148.777 | -0.089 | 0.534 | 0.716 |  | 1148.777 | -0.010 | | 0.944 | | 1.000 | |  |
| WNK4 | 5.444 | 0.085 | 0.538 | 0.719 |  | 5.444 | -0.092 | | 0.519 | | 1.000 | |  |
| CD2 | 1931.509 | 0.063 | 0.542 | 0.723 |  | 1931.509 | -0.067 | | 0.540 | | 1.000 | |  |
| VWF | 155.675 | -0.077 | 0.577 | 0.749 |  | 155.675 | -0.292 | | 0.040 | | 0.923 | |  |
| TRPC3 | 18.313 | 0.065 | 0.653 | 0.803 |  | 18.313 | 0.034 | | 0.815 | | 1.000 | |  |
| UTS2 | 289.663 | 0.058 | 0.675 | 0.816 |  | 289.663 | 0.092 | | 0.495 | | 1.000 | |  |
| PTPN1 | 1893.774 | 0.021 | 0.705 | 0.838 |  | 1893.774 | -0.044 | | 0.471 | | 1.000 | |  |
| CYP11B1 | 4.112 | 0.047 | 0.738 | 0.860 |  | 4.112 | 0.050 | | 0.714 | | 1.000 | |  |
| SLC12A3 | 10.270 | 0.036 | 0.801 | 0.899 |  | 10.270 | 0.249 | | 0.087 | | 1.000 | |  |
| CCR5 | 545.676 | 0.031 | 0.809 | 0.904 |  | 545.676 | -0.075 | | 0.577 | | 1.000 | |  |
| MAPK10 | 3.459 | -0.019 | 0.898 | 0.952 |  | 3.459 | -0.294 | | 0.040 | | 0.923 | |  |
| MEX3C | 1117.059 | -0.008 | 0.923 | 0.965 |  | 1117.059 | 0.078 | | 0.367 | | 1.000 | |  |
| ALOX12 | 395.002 | 0.009 | 0.924 | 0.965 |  | 395.002 | -0.061 | | 0.561 | | 1.000 | |  |
| ERAP1 | 2483.285 | 0.007 | 0.933 | 0.970 |  | 2483.285 | 0.043 | | 0.623 | | 1.000 | |  |
| HSD11B2 | 3.747 | -0.008 | 0.957 | 0.979 |  | 3.747 | -0.117 | | 0.410 | | 1.000 | |  |
| RETN | 174.766 | 0.000 | 0.999 | 0.999 |  | 174.766 | 0.001 | | 0.996 | | 1.000 | |  |
| CEN, centenarians; F1, first generation of offspring; F1SP; spouses of F1; *p value of CEN vs. F1SP <0.05 | | | | | | | | | | | | | |
| | **Supplemental table 4. Expression comparisons of genes associated with kidney function.** | | | | | | | | | | | --- | --- | --- | --- | --- | --- | --- | --- | --- | --- | |  |  | **CEN vs. F1SP** |  |  |  |  | **F1 vs. F1SP** |  |  | | **Gene name** | **Base Mean** | **log2FoldChange** | **p value** | **adjusted p** |  | **Base Mean** | **log2FoldChange** | **p value** | **adjusted p** | | LRRC16A* | 52.358 | -0.788 | 0.000 | 0.000 |  | 52.358 | -0.343 | 0.008 | 0.754 | | DIP2C* | 70.884 | -0.469 | 0.000 | 0.004 |  | 70.884 | -0.277 | 0.031 | 0.923 | | SLC28A2* | 10.091 | -0.304 | 0.018 | 0.091 |  | 10.091 | -0.208 | 0.120 | 1.000 | | ASL* | 430.621 | 0.244 | 0.005 | 0.043 |  | 430.621 | 0.203 | 0.032 | 0.468 | | CKM* | 3.878 | 0.326 | 0.024 | 0.109 |  | 3.878 | 0.055 | 0.706 | 1.000 | | [ARG1*](http://www.ncbi.nlm.nih.gov/gene/383) | 195.872 | 0.282 | 0.044 | 0.172 |  | 195.872 | 0.127 | 0.371 | 0.468 | | [CKMT2](http://www.ncbi.nlm.nih.gov/gene/1160) | 10.296 | -0.275 | 0.056 | 0.184 |  | 10.296 | -0.045 | 0.759 | 1.000 | | GABRR2 | 80.058 | -0.196 | 0.173 | 0.365 |  | 80.058 | -0.215 | 0.139 | 1.000 | | MRPS30 | 258.719 | 0.075 | 0.330 | 0.542 |  | 258.719 | 0.015 | 0.855 | 1.000 | | SLC7A9 | 5.772 | -0.108 | 0.435 | 0.636 |  | 5.772 | -0.014 | 0.920 | 1.000 | | OAT | 642.736 | 0.052 | 0.474 | 0.668 |  | 642.736 | 0.099 | 0.208 | 1.000 | | CEP89 | 86.936 | 0.030 | 0.672 | 0.815 |  | 86.936 | 0.099 | 0.192 | 1.000 | | GAMT | 59.476 | -0.024 | 0.815 | 0.907 |  | 59.476 | -0.033 | 0.760 | 1.000 | | GATM | 21.700 | -0.030 | 0.831 | 0.915 |  | 21.700 | 0.200 | 0.167 | 1.000 | | WDR1 | 7611.075 | 0.079 | 0.192 | 0.389 |  | 7611.075 | 0.053 | 0.421 | 1.000 | | ALDH16A1 | 974.442 | 0.123 | 0.110 | 0.278 |  | 974.442 | 0.001 | 0.990 | 1.000 | | ARID1B | 1524.045 | -0.119 | 0.094 | 0.253 |  | 1524.045 | -0.003 | 0.974 | 1.000 | | VSTM4 | 38.003 | -0.123 | 0.354 | 0.565 |  | 38.003 | -0.055 | 0.663 | 1.000 | | ABCG2 | 10.154 | -0.157 | 0.277 | 0.488 |  | 10.154 | -0.043 | 0.767 | 1.000 | | SLC2A9 | 101.087 | 0.088 | 0.401 | 0.608 |  | 101.087 | 0.170 | 0.127 | 1.000 | | TMEM18 | 262.211 | -0.080 | 0.136 | 0.317 |  | 262.211 | -0.053 | 0.365 | 1.000 | | SEMA5A | 14.586 | -0.165 | 0.257 | 0.466 |  | 14.586 | 0.250 | 0.083 | 1.000 | | GORASP2 | 602.761 | 0.047 | 0.434 | 0.635 |  | 602.761 | -0.003 | 0.965 | 1.000 | | METTL6 | 128.509 | -0.070 | 0.275 | 0.486 |  | 128.509 | 0.017 | 0.802 | 1.000 | | ARHGAP26 | 5687.160 | -0.124 | 0.306 | 0.520 |  | 5687.160 | -0.017 | 0.893 | 1.000 | | MYO18B | 5.939 | 0.160 | 0.221 | 0.426 |  | 5.939 | 0.119 | 0.342 | 1.000 | | CNTN4 | 6.414 | 0.026 | 0.860 | 0.931 |  | 6.414 | -0.079 | 0.587 | 1.000 | | CEN, centenarians; F1, first generation of offspring; F1SP; spouses of F1; *p value of CEN vs. F1SP <0.05 | | | | | | | | | | | | | | | | | | | | | | | |
